# Distributed Cognition and the Experience of Presence in the Mars Exploration Rover Mission

**DOI:** 10.3389/fpsyg.2021.689932

**Published:** 2021-06-21

**Authors:** Dan Chiappe, John Vervaeke

**Affiliations:** ^1^Department of Psychology, California State University, Long Beach, CA, United States; ^2^Department of Psychology, University of Toronto, Toronto, ON, Canada

**Keywords:** shared presence, joint agency, distributed cognition, Mars exploration, embodied cognition

## Abstract

Although research on presence in virtual environments has increased in the last few decades due to the rise of immersive technologies, it has not examined how it is achieved in distributed cognitive systems. To this end, we examine the sense of presence on the Martian landscape experienced by scientific team members in the Mars Exploration Rover (MER) mission (2004–2018). How this was achieved is not obvious because the sensorimotor coupling that typically underlies presence in mundane situations was absent. Nonetheless, we argue that the Three-Level model can provide a framework for exploring how presence was achieved. This account distinguishes between proto-presence, core-presence, and extended-presence, each level dependent on being able to respond effectively to affordances at a particular level of abstraction, operating at different timescales. We maintain that scientists' sense of presence on Mars involved core-presence and extended-presence rather than proto-presence. Extended-presence involved successfully establishing distal intentions (D-intentions) during strategic planning, i.e., long term conceptual goals. Core-presence involved successfully enacting proximal intentions (P-intentions) during tactical planning by carrying out specific actions on a particular target, abstracting away from sensorimotor details. This was made possible by team members “becoming the rover,” which enhanced their ability to identify relevant affordances revealed through images. We argue, however, that because Mars exploration is a collective activity involving shared agency by a distributed cognitive system, the experience of presence was a collective presence of the team through the rover.

## Introduction

The development of immersive digital technology has created opportunities for interacting with remote or simulated virtual environments across a wide range of domains (Goldberg, 1999). The central goal has been to design tools that produce a sense of “presence,” a sense of “being there,” in a technologically-mediated environment, on the assumption that this enhances performance (Cummings and Bailenson, 2016; Grassini et al., 2020; Toet et al., 2020). This has led to metrics for assessing presence (e.g., Grassini and Laumann, 2020) as well-research aimed at clarifying the concept (e.g., Sheridan, 1992; Lee, 2004; Pillai et al., 2013) and the psychological and neurological processes that underlie it (e.g., Zahoric and Jenison, 1998; Seth et al., 2012; Riva and Waterworth, 2014).

The research on presence has largely focused on individual processes, ignoring research on distributed cognition that has simultaneously come to fruition in cognitive science (Hutchins, 1995; Heersmink, 2015; Slors, 2020). Distributed cognition claims that in certain types of collective action, the proper unit of analysis for cognitive processes is that of a system made up of multiple interacting technical and non-technical agents with heterogeneous properties (Milkowski et al., 2018). This is particularly the case in domains where people interact with massively complex objects and situations. It is unclear, however, how research that explains how presence is achieved by people working alone can scale-up to explain how it is achieved by a distributed cognitive system. A more complete explication of presence may therefore require considerable theoretical development. We address this issue by proposing a framework for guiding research into how presence is achieved by the socio-technical system exploring the Martian surface.

Mars exploration is conducted using technology that includes rovers such as *Spirit* and *Opportunity* in the Mars Exploration Rover mission (MER; 2004-2018), *Curiosity* in the Mars Science Lab mission (MSL; 2012-present), and *Perseverance* in the Mars 2020 mission. Although it is a technologically-mediated activity, ethnographers using open-ended interviews to query scientists about their experiences working with rovers report that team members have a collective sense of presence on Mars (Clancey, 2012; Vertesi, 2015). This is so despite the fact that actions are carried out millions of miles away, such that the normal sensorimotor contingencies that help to ground presence in mundane situations are unavailable (Chiappe and Vu, 2019). We explain how this is possible using Riva and Waterworth's 2014 Three-Level model, which distinguishes proto-presence, core-presence, and extended-presence. Presence at each of these levels depends on being able to respond effectively to affordances at different levels of abstraction and at different timescales. We argue that scientists' sense of presence is best described as core-presence and extended-presence, facilitated by “becoming the rover,” rather than proto-presence, and we outline how the model needs to be revised to account for the fact that Mars exploration is a collective activity that involves shared agency by a distributed cognitive system (Chiappe and Vervaeke, 2020). This is done by describing how the communication processes and rituals involved serve to produce genuinely shared, group-level intentions. Although our scheme relies for evidence on reports by participant-observer ethnographers, this framework is testable and can guide further research into this topic.

## The Mars Exploration Distributed Cognitive System

**Figure 1:**
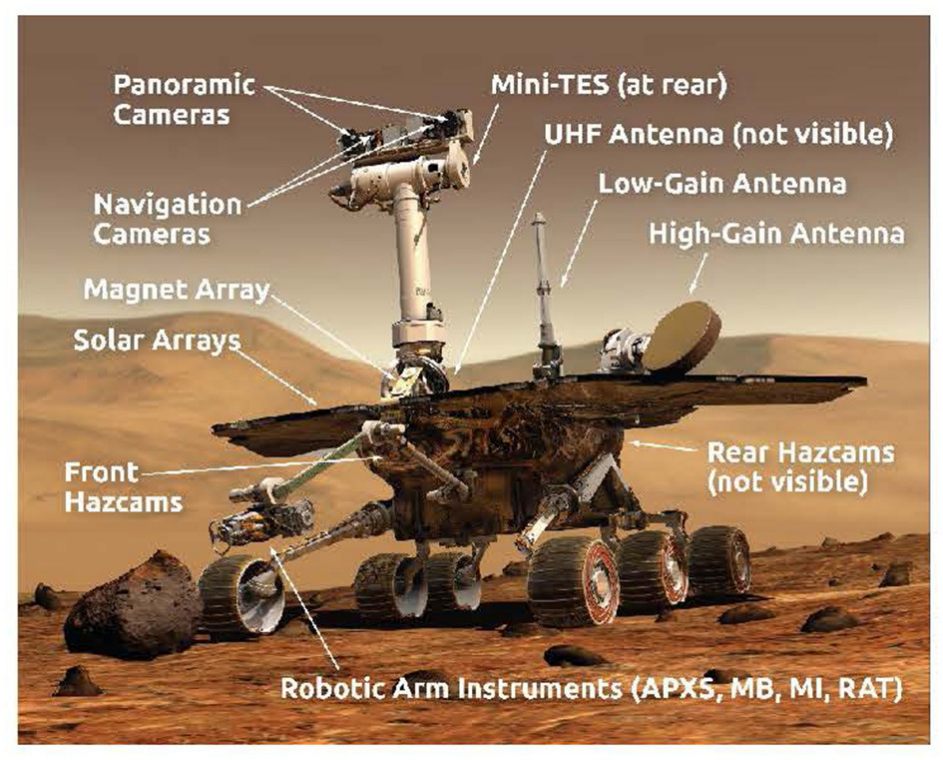
MER rover and tools. Image credit: NASA/JPL-Caltech.

Before discussing the topic of presence, it is important to describe how Mars rover missions operate. Our focus will be mainly on the MER mission because it has received extensive ethnographic work (i.e., Clancey, 2012; Vertesi, 2015), and the remarkable success of this mission has informed the way subsequent rover missions are conducted. The six-wheeled rovers *Spirit* and *Opportunity* were equipped with a suite of tools that included nine cameras, three spectrometers, and a rock abrasion tool (see Figure 1 and Appendix for brief description of tools). The tools were designed to complement one another by providing mutually-supporting evidence from different sources to establish scientific claims, enabling teams of scientists to do field geology remotely. The rover tools, however, were not controlled directly by the scientists. Instead, scientists developed strategic and tactical plans for drives and observations, and engineers used these to develop instructions that were sent to the rovers once per sol.

The MER team members can be broadly divided into science and engineering disciplines, with a high degree of specialization within each of these domains. The dozens of scientists working the mission, for example, were divided into the following Science Theme Groups (STGs): (1) Geology, (2) Mineralogy and Geochemistry, (3) Rocks and Soil, and (4) Atmospherics (Squyres, 2005). Scientists were responsible for determining which instruments to apply in particular situations to achieve their scientific objectives, as well as interpreting the data returned from the rovers each sol. Engineers were involved in actually operating the technology. Among them, however, there was also much specialization and division of responsibility. Each tool, for example, had instrument specialists, i.e., payload uplink leads devoted to compiling commands for a rover's upcoming daily operation, and payload downlink leads, who monitored the health and status of the instrument. There were also Rover Planners responsible for planning a rover's drives (Clancey, 2012).

Despite the fact that the mission was made up of many individuals pursuing highly specialized tasks, the MER team managed to function in a unified manner to explore Mars. This is a deliberate result of the way the decision-making process was structured by the Principal Investigator (Squyres, 2005). Although each STG was composed of members that worked independently to address their scientific objectives, they also gathered to collectively discuss scientific results and to engage in strategic and tactical planning. The strategic planning meetings on MER, known as the weekly “End of Sol” meetings, established the team's scientific goals for the upcoming weeks (or months for longer campaigns), and served to constrain tactical activities. Tactical planning, in contrast, involved scientists deciding upon activities for the next sol in the Science Operation Working Group (SOWG) meetings (Clancey, 2012). Using imagery and instrument data returned from the previous sol, they collectively decided which targets to analyze, or which direction to travel. They took into account the strategic goals as well as what engineers determined was safe to do given the current status and resources of the rover and its tools.

A unity of purpose and complementarity of effort was achieved through consensus-based operations. This is most clearly illustrated in the SOWG meetings, which had a highly ritualized structure designed to produce consensus among science team members. At the start of the meeting, scientists were presented with the “skeleton,” a file that lists all the constraints, including when the rover needed to recharge its batteries, when it had to communicate with Earth based on satellite passes, and what times were available for the science team to request observations. It provided a frame into which all planned activities must fit. Next, the SOWG Chair opened the meeting for discussion, allowing each STG to propose observations. All proposals had to be justified based on their scientific relevance, but had to take into account the “bio-economics” of the rover and the resources available. Any team member could comment on any proposed observation. The back-and-forth discussions ensured observations were relevant to testing hypotheses in the most efficient manner possible. Importantly, the SOWG Chair ensured all team members were satisfied with the plan prior to finalizing it by going around the room asking if each group was “happy” with the plan (Vertesi, 2015).

Figuring out Mars at the planetary scale exceeds the capacity of any one individual because it is what Morton (2013) calls a “hyperobject,” a multidimensional object massively distributed across time and space. The best way to get a grip on the features of such an object is through a distributed cognitive system, which MER has all the hallmarks of being. Distributed cognition claims that cognitive processes transcend the internal operations of individuals to include the interactions that take place between multiple agents, some of which are technological, like rovers (Hutchins, 1995; Heersmink, 2015; Slors, 2020). According to Sutton (2010), the creation of a distributed cognitive system rests on the *complementarity* of the functions of the different agents of the sociotechnical system. Cognitive systems can be made up of components with very different properties and functions, provided that they work together to create an intelligent system that functions with a unified purpose, i.e., they provide “collective and complementary contributions to flexible thinking and acting” (Sutton, 2010, p. 194). In MER, the consensus-based operations allowed for creativity and critical thinking that bound the group together, working like one mind with myriad sources of good ideas. Clancey (2012) reports one team member saying, “You try to foster an environment in which ideas of all kinds are encouraged and respected, but in the end, the best ideas win. If you can do that, then…it's like having one guy with an IQ of 10,000” (p. 147).

## How Is Presence on Mars Achieved?

### Problematizing Presence on Mars

The ethnographic reports reveal that scientists and engineers working on MER came to have a sense of presence on Mars; they were present as the rover on the Martian surface (Clancey, 2012). “Presence” refers to “the subjective sense of reality of the world and of the self within the world” (Seth et al., 2012, p. 1). It is the sense of “being there,” in an environment that feels real. When the sense of presence is mediated through technology it is referred to as ‘telepresence,' the sense of being in an environment without awareness of the technology that is mediating that experience (Sheridan, 1992; Lee, 2004; Pillai et al., 2013). The sense of telepresence on Mars is evident in how team members described their experiences. The ethnographic record is full of statements like “we are 4 m from the outcrop we want to image” (Vertesi, 2015), “we have arrived at Endurance Crater,” and “it's below our feet,” referring to Lakebeds at Gusev (Clancey, 2012). In another typical statement, Jim Rice, an astrogeologist, said “I put myself out there in the scene, the rover, with two boots on the ground, trying to figure out where to go and what to do” (Clancey, 2012, p. 100). Likewise, the PI Squyres said, “if you had told me that *we* were going to climb to the summit of Husband Hill, and get to Home Plate, then get down into Endurance Crater, then Victoria Crater…I wouldn't have believed it” (Clancey, 2012, p. 101). Nathalie Cabrol, a planetary geologist, said “if we spend more time here, we're not making it to the point where it's the safe haven for winter, and then were going to die” (Clancey, 2012, p. 131).

The experience of telepresence on Mars is perplexing because it does not fit the profile of most telepresent experiences. In typical cases where it is experienced, a teleoperation mode of control is employed. In teleoperations, the operator controls a remotely-located robot often through a joystick, issuing commands that are enacted through actuators and receiving real-time, continuous feedback from sensors (e.g., Minsky, 1980; Sheridan, 1992; Toet et al., 2020). When combined with displays providing an immersive interface, the operator can feel present in the remote environment (Cummings and Bailenson, 2016). An example is picking up a distant object by using one's hand to manipulate a joystick that moves a robotic arm. In this type of mediated action, the distal tool is incorporated into the body schema, creating a shift in peripersonal space (Riva and Mantovani, 2012). Indeed, near space and far space come to be centered on the distal tool. This leads to the experience of spatial presence in the remote environment. It locates the self in a distal place from which it can monitor its actions (Lee, 2004).

This does not, however, capture the way that telepresence was achieved by Mars scientists. This is because they did not directly manipulate proximal tools that controlled the rover's actions. Instead, engineers controlled the rovers, though they did not employ teleoperations due to the lengthy time delays in sending and receiving signals. Instead, they used “batch programming,” where a set of commands referred to as “the sequence” was uploaded to the rovers once per sol, stored in flash memory, and executed at designated times (Mishkin et al., 2006). The feedback was thus discontinuous, relying on static imagery to make decisions and assess the results of previous actions. Of course, scientists were involved in the process—they identified targets to be investigated and what actions to take on those targets. To do so, they worked with images and instrument readings. They also used software with which they could select images to specify the targets that needed to be examined. This information was then used by engineers to generate the daily command sequence. Moreover, scientists did not typically see images of the rover's tools as they interacted with distal objects. Images were usually focused on features of the Martian surface itself. As a result, the conditions that typically lead to the incorporation of a distal tool were not present (Martel et al., 2016). It is therefore perplexing how the shift of perspective to the Martian surface took place.

According to Mindell (2015, pp. 184–185) “scientists feel they are working on Mars because their perceptions, their teamwork, the interplanetary system, and the rovers make a kind of cognitive sense. The team on the ground sees things in the world, considers the data and imagery, makes decisions, sends commands to the rovers, and sees the results of their actions.” Indeed, Mindell (2015) claims the lengthy delays allowed for deep immersion in the data and intense discussions among team members about what they mean and how to proceed to further test scientific hypotheses. We maintain, however, that a much more detailed account is needed to capture the phenomenology of team members feeling present on Mars, and how it is possible despite the absence of the sensorimotor loop that normally accompanies presence.

### The Three-Level Model of Presence

Many analyses have shown that “presence” is a multi-dimensional concept (e.g., Draper et al., 1998; Lee, 2004; Carlson et al., 2017). An approach that is useful for understanding the complexity of the experience of presence in Mars exploration is the Three-Level model of Riva and Waterworth (2014) Triberti and Riva, 2016). According to this model, presence is intimately connected with agency and intentional action. This is supported by empirical research showing, for example, that sense of presence is greater in virtual game environments when people can act in the environment instead of merely being spectators (Havranek et al., 2012). Following Zahoric and Jenison (1998), the Three-Level model holds that presence is the feeling of inhabiting an environment that arises when one has an optimal grip on relevant affordances. We can sense deviations in presence in tasks we are engaged in and take steps to regain optimal grip (Chiappe and Vu, 2019). Although presence is usually experienced as a unitary phenomenon, it is made up of different layers that can be differentiated in certain situations. A maximal sense of presence arises when all three layers coincide. Indeed, operators can experience flow under these conditions (Triberti et al., 2016, 2021).

Specifically, the Three-Level model distinguishes between proto-presence, core-presence, and extended-presence, each related to one of the three levels of the self that Damasio (2011) identifies. Each level of presence involves enacting intentions that reflect environmental opportunities for action. Without this dovetailing of intentions with relevant features of the environment, we would not have an optimum grip, and we would not experience presence. As they say, “I am present in a real or virtual space if I manage to put my intentions into action (enacting them). Feeling variations in the sense of presence, one can monitor his own actions and tune his activity accordingly” (Triberti and Riva, 2016, p. 2). However, there are different kinds of intentions associated with each of the three levels of presence.

### Proto-Presence

Proto-presence reflects the degree of perception-action coupling as we are engaged in interactions with objects (Riva and Waterworth, 2014). Micro-movements of the body have to be tightly coupled to relevant features of objects to be effective. For example, the hand has to adjust its orientation and grip to match the specific features of the object being grasped. Proto-presence results from the operation of the *proto-self*, whose function is to dynamically track the current physical state of the body as it responds to environmental changes. This includes tracking the status of the internal milieu, including the viscera (i.e., interoception), the musculoskeletal position of the body and its limbs (i.e., proprioception), as well as input from the external senses (exteroception) as an action is unfolding. According to Damasio (2011), tracking the current state of the body and its location in the world is crucial for establishing a situated perspective.

The intentions that underlie proto-presence are what Pacherie (2006) calls “motor intentions” (M-intentions). These occur along with an action, providing fine-grained guidance and control, operating over elemental timescales (i.e., 10–500 ms). Their content is sensorimotor, as sensory information about the affordances present in the object are used to select appropriate motor responses. Proto-presence thus results from our ability to monitor whether the body is correctly carrying out its M-intentions by determining whether they are having the expected interoceptive and exteroceptive effects. When motor responses correctly predict consequences at the elemental timescale, the result is proto-presence.

### Core-Presence

This is presence accompanying actions specified at a higher level, abstracting from specific sensorimotor details. For example, perceiving that one needs to get out of the way of an object moving in one's direction and doing so successfully without focusing on the specific movements involved is core-presence. Core-presence results from the operations of the *core-self* which has the task of creating a perception of salient objects. The core-self integrates various sensory impressions into a stable percept, an ability tuned toward perceiving affectively-relevant objects in the environment (Damasio, 2011). It tracks changes in affect and when these are significant, it focuses attention on perceptual objects causing those changes and initiates appropriate actions. Thus, it is a second-order tracking of the objects and events that are affecting the proto-self.

The intentions that underlie core-presence are what Pacherie (2006) calls “present-directed intentions” (P-intentions). These intentions, also referred to as “proximal intentions,” initiate an action in a particular context and sustain it until it is complete. They provide high-level guidance as the action unfolds, tracking the action as a whole and monitoring for collateral effects. If the action is having unwanted side effects, a high level command is issued to eliminate these effects by influencing the formation of M-intentions. P-intentions are formulated in the situation in which they are enacted, i.e., while in perceptual contact with the relevant objects, and are therefore anchored to particular situations. They have indexical content, i.e., doing *this* action on *this* particular object, and operate over integrative timescales (i.e., 500 ms to 3 s).

### Extended-Presence

Extended-presence arises when one successfully formulates long term, conceptually-articulated goals. Extended-presence is greatest when those goals are attainable. According to Riva and Waterworth (2014), extended-presence relies on the operation of the *autobiographical self*. The autobiographical self is able to set goals not necessarily related to the here and now, ones consistent with narratives that define its identity. It allows us to plan activities and imagine possible future situations. Although the goals are often defined abstractly, they have to reflect possibilities for action in the world as well as our self-narratives.

The intentions that underlie extended-presence are what Pacherie (2006) calls “distal- intentions” (D-intentions). D-intentions serve as terminators of practical reasoning about *ends*, as they involve arriving at a decision about a course of action that will be undertaken. They also prompt practical reasoning about *means* by generating a plan for achieving the goal in question. The content of shared D-intentions is conceptual, i.e., offering a general description of a type of action to be carried out, and they are formed prior to actions and separate from the situation in which a concrete action will actually unfold. These intentions operate over narrative timescales (i.e., > 3 s).

According to the Three-Level model, the intentions that underlie the three types of presence form an action cascade. D-intentions generate P-intentions when relevant affordances are identified. P-intentions, in turn, generate M-intentions, though the latter also depend on the details of the environment where the action is taking place. In other words, D-intentions have to dovetail with distal affordances in that it must be possible within the milieu in question to achieve the general goal (Triberti and Riva, 2016). However, to reduce the discrepancy between the current state of the world and these abstract intentions, D-intentions have to be transformed into P-intentions. These are consistent with the D-intentions insofar as they help to achieve them, but they are more situated with respect to environmental affordances; target objects have to be perceptually present. P-intentions, though formed while in perceptual contact with objects, define the task to be carried out in a way that abstracts away from the specific movements. But, as proximal affordances are approached, motor affordances reveal themselves and help to determine the best M-intentions for the situation.

The experience of presence is the strongest, and a feeling of flow can be attained, when all three levels are focused on the same external situation (Triberti et al., 2021). When each layer is stimulated by non-overlapping content, however, the overall sense of presence is reduced. In normal circumstances, proto-presence and core-presence are focused on the same object. But if one's mind wanders, one's autobiographical self may be focused on other things, diminishing the overall sense of presence.

### The Three-Level Model and Mars Exploration

In what follows, we apply the Three-Level model to the case of the exploration of Mars through the *Spirit* and *Opportunity* rovers. We argue that although proto-presence was not possible, we can characterize the experience of the Mars exploration team as involving core- and extended-presence. However, the model needs to be expanded to capture the fact the timescales involved are typically much longer than in mundane situations, and the fact that the experience is that of a *collective* presence, because the agency that is involved is a shared agency. No one acts alone in carrying out activities on the surface of Mars. The activities reflect the intentions of a distributed cognitive system (Chiappe and Vervaeke, 2020).

### Extended-Presence on Mars

Extended-presence involves the formation of D-intentions, ones that reflect conceivable opportunities for action in the environment. They prompt reasoning about a course of action that can be undertaken in the future. An example of a D-intention was for *Opportunity* to explore Victoria Crater. The intention was to enter the 800 m wide crater and study the layers that form the cliff face, gathering images and spectrographic readings to determine how the layers of bedrock were built-up millions of years ago (Vertesi, 2015). This D-intention prompted deliberation about the means for carrying out this plan, including determining exactly what route to take into the crater. To facilitate the decision, the team opted to drive around the rim of the crater taking Pancam images to identify potential ingress points.

The D-intentions guiding the actions of the MER rovers, however, were not individual intentions, they were shared intentions—ones belonging to the team. As a result, extended-presence on Mars was a *collective* extended-presence. Shared intentions are intentions about what the “*we*” plans to do, reflecting the will of the group (Tollefsen, 2014). Intentions can be attributed to a group if they are arrived at through free and open discussion between all members, and the ultimate decisions reflect everyone's input. If this condition is met, it produces an intention “that is ‘subjectless' not because it presupposes the participants adopt a neutral perspective…but rather because the process of deliberative discourse itself neutralizes the subject-centered contributions of the participants” (Bacon, 2012, p. 134). In MER, shared D-intentions were arrived at during strategic planning meetings, the weekly “End of sol” meetings led by a Long Term Planning Lead. Strategic meetings also included each of the four STGs, as well as engineers who provided input on the health and status of the rover and its instruments. During these meetings, the team had to arrive at a consensus regarding the activities of the rover in the coming weeks or months. The intentions described the goals in general terms, as specific targets were typically selected for investigation during tactical planning meetings. Consistent with the Three-Level model of presence, the process of arriving at D-intentions operated over narrative time scales and they were formed prior to the joint action.

**Figure 2:**
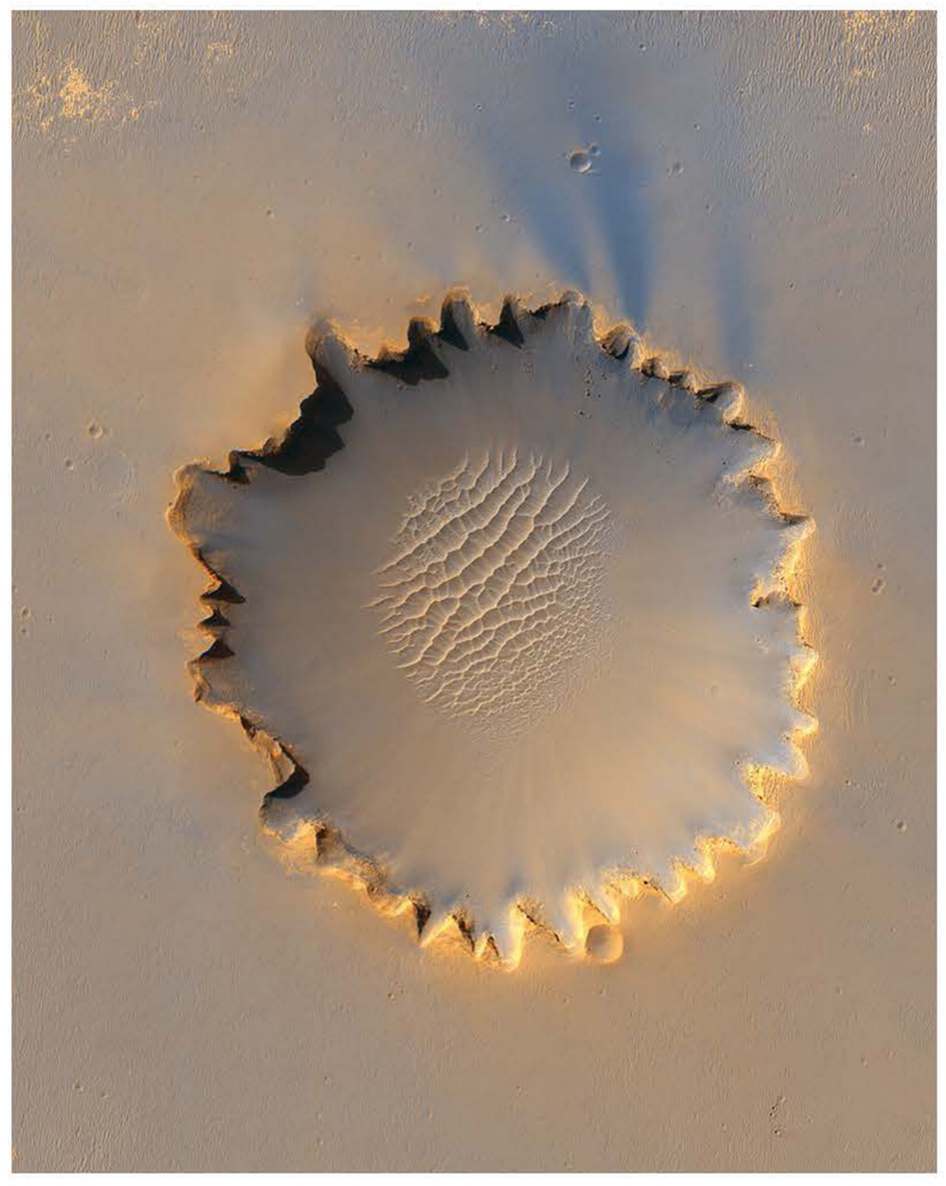
HiRISE orbital image of Victoria Crater taken by Mars Reconnaissance Orbiter. Image credit: NASA/JPL.

The establishment of a collective sense of extended-presence on Mars was facilitated by scientists interacting with images of the Martian region they were interested in exploring. Indeed, doing field geology on Mars without such images is impossible. Specifically, orbital images of a region were often used to discuss potential D-intentions. For example, such images of Victoria Crater were drawn on to indicate potential locations for collecting Pancam images of its walls, as well as to identifying potential paths for exploring the region (see Figure 2). These were initially labeled “draft,” allowing for input from all participants. The maps were not static, as they were updated based on ongoing discussions among team members. When more information was needed they were labeled with question marks (Vertesi, 2015). The annotations on the images also captured when consensus regarding D-intentions had been achieved.

The Three-Level model holds that extended-presence relies on the autobiographical self. This is the self that has a trans-temporal identity. It allows us to plan activities beyond the here and now, and therefore to imagine possible future situations. This is the self that forms the center of “narrative gravity” (Dennett, 1991). This sense of self is constituted by the narratives that define who the individual is. People consider intentions that form a coherent continuation of the story they tell about themselves (Velleman, 2007). Narratives can thus play a role in self-governance, helping to provide stability and coherence to intentions, thereby increasing extended-presence.

In the case of long-standing groups, the narratives involved in constituting an identity are what Gallagher and Tollefsen (2019) call “we-narratives.” These are narratives about what a group has done, is doing, or will do. They serve to define the shared identity of members in the group, specifying its structure, goals and collective values. We-narratives also include what their mission is and what the group's plans are for the future. They provide an important backdrop against which deliberation and intention-formation take place. Importantly, intentions that do not cohere with the narrative are unlikely to drive collective action. Intentions that are seriously considered are ones that are consistent with the story the group tells about itself.

In MER, we-narratives included narratives about what the group was trying to accomplish on Mars. Shared D-intentions therefore had to be relevant to addressing the goals of the mission, which were the domain of different STGs. These were: (1) assessing the past habitability of Mars by analyzing the geological and chemical features of rocks and soils for evidence of past water activity; (2) studying the Martian climate by analyzing the temperature profile of the atmosphere; (3) identifying the geological history of Mars by examining the role of wind, water, tectonic, and volcanic activity in modifying its surface; and (4) preparing for human missions to Mars by identifying potential resources and hazards (Squyres, 2005). Although the goals of some STGs took priority at some points, the we-narrative defining the group included a commitment to taking care of everyone's science in the long run. This fostered a spirit of compromise and cooperation among science team members.

Other essential features of the we-narratives in MER included a commitment to a flattened social hierarchy. Individuals were obligated to be open to revising their beliefs in response to reasoned discussion with others. During planning meetings, MER team members collectively examined each other's views prior to arriving at a decision. True to the flattened hierarchy, discussions did not appeal to authority, seniority, or other factors pertaining to the identity of the members. Instead, the factors that were considered included matters of scientific relevance. Engineering factors were also legitimate issues to discuss during deliberation. This included, for example, the health of the rover and its instruments, and the basic need to keep the rover alive so that the mission could continue.

### Core-Presence on Mars

Core-presence is the presence one experiences when actions are effectively carried out on objects, abstracting from micro-movements and specific sensorimotor details. Core-presence arises through the successful enactment of P-intentions. In MER, a P-intention would be, for example, to take an infrared reading by applying the mini-TES tool to a specific soil sample, or using the rock abrasion tool (RAT) to grind the surface of a particular rock. Core-presence involves tracking actions to make sure they are proceeding as intended and involves monitoring for unwanted side-effects. In mundane situations, this happens within an integrative timescale while the action is unfolding. Although in the case of Mars exploration actions were tracked to make sure intentions were fulfilled, the feedback was not available until data was downloaded, thus operating within narrative timescales. In particular, instrument experts analyzed downloaded data from the instruments to verify that the observations were reliable and met scientific requirements (Mishkin et al., 2006). Likewise, before images were passed along to the scientists, they had to be inspected and calibrated by individuals trained for that purpose. The image calibrators were trained to detect anomalies in images and to work with software to “clean up” images by removing sources of variation such as time of day, dust patterns, etc. This served to produce a standardized picture that could be used by the scientists (Vertesi, 2015). If the images or instrument actions were deemed to be problematic, this was reported to the scientists at the start of the next SOWG meeting. Repeating the observations then became a priority for the next sol.

As with D-intentions, P-intentions on MER were shared intentions. This is because they were formulated during SOWG meetings, which made use of consensus-based operations; all participants contributed to the daily plan and could challenge any proposed observation, and all had to indicate their approval before it was finalized. This ritual of asking everyone if they were satisfied with the plan required “team members to express their continued commitment to the mission and reminds them that in doing so they are all complicit in the day's activity plan” (Vertesi, 2015, p. 45). Shared P-intentions were constrained by strategic plans, i.e., shared D-intentions, as well as by the current resources available to the rovers. Engineers provided scientists with a framework during which observations could take place, taking into account, for example, when a rover had to “nap” to recharge its batteries, and when it had to pause activities to communicate with Earth through satellites. During the nominal mission (first 90 sols), SOWG meetings determined a plan for the rover's actions the next sol, enacting a one-sol turnaround cycle of operation. Each sol's plans reflected feedback regarding the previous sol's activities. This cycle kept scientists engaged and maximized the scientific return of the mission but was grueling because team members had to live on Mars time, the Martian day being 40 min longer (Squyres, 2005).

Core-presence involves interactions with perceptible objects. In the case of exploration through rovers, however, scientists and engineers were not in direct perceptual contact with the Martian features they interacted with. Instead, image-work played a crucial role in doing remote field science and in achieving consensus on P-intentions. Images were projected on the screens during the SOWG meetings using the Science Activity Planning software so that all team members could comment on the proposed observations (Clancey, 2012). For example, scientists put red dots on rocks in a Hazcam image to indicate which should be subjected to mini-TES infrared “stares.” These images were also shared with engineers to allow them to finalize the sequence of commands uploaded to the rovers. In the course of developing P-intentions, names were given to targets to avoid ambiguity and to ensure the correct target was interacted with. Naming practices therefore helped to convert the general plan into an action on a particular object, converting a D-intention to a P-intention. As (Vertesi, 2015, p. 120) says, “Names ground and document both the team's interactions, in terms of coming to agreement on a target location, and the rover's interactions, in terms of performing the requested observations on Mars.”

**Figure 3:**
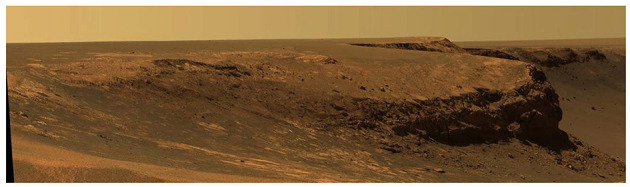
Pancam image of Cape Verde on Victoria Crater taken by *Opportunity*. Image credit: NASA/JPL/Cornell.

The images used in the MER mission varied in scale. At the broadest scale were large panoramic photographs taken by the Pancams (e.g., Figure 3), and at the smallest scale were images taken by the microscopic imager (MI). The large panoramas were often laid out on tables so that scientists could collectively examine details of the landscape in front of the rover, or were looked at as either 2D or 3D images on computers, both formats being able to induce a sense of core-presence (Baños et al., 2008). These images established a “seeing from” perspective—-providing a location from which the viewing was taking place, centered on the position of the rover (Ihde, 1990). This perspective changed as the rover traveled across the surface. As a result, the landscape was revealed as a sequence of vistas that gradually uncovered certain features of the terrain while occluding others (Ingold, 2011). When looking at panoramic images, MER team members interpreted them as wayfarers who had to sustain themselves through interactions with the environment as they traveled along paths in the Martian landscape. As they did so, they experienced horizons, openings into which movement was possible.

There is a phenomenological difference between looking at an image for aesthetic purposes and using an image to sustain an engaged remote activity. Although aesthetic images were published (see Bell, 2006, *Postcards from Mars*), these were mostly for raising awareness of the mission among the public. Scientists and engineers, however, used images to either plan rover movements or to deploy instruments to answer scientific questions. In the case of Mars exploration what images did was extend the affordance space so that opportunities for action by the rovers on Mars would present themselves to team members on Earth. Affordances are possibilities for action provided to us by the environment, and they are defined relative to abilities available in a certain kind of practice, a “form of life” (Gibson, 1979; Rietveld et al., 2018). In the case at hand, affordances were defined relative to the socio-technical skills for doing remote science provided by the MER distributed cognitive system that included scientists, engineers, and the current state of the rover and its tools.

When looking at images, what MER team members saw was what they could enact through the rover, and they anticipated results that would be evident in subsequent imagery. Furthermore, they sought an optimal grip on the landscape of affordances revealed by these images. Although at any given time there were many possible courses of action, the team was drawn toward an optimal grip on *relevant* affordances (Rietveld et al., 2018). Failure to do so would have led to a loss of a sense of presence according to the Three-level model because it claims that the sense of presence is dependent on having such a grip. Indeed, team members could sense when the rover was not suitably placed in the Martian terrain to meet science and engineering goals, and they took steps to remedy the situation. For example, based on an initial image of Bonneville Crater, Squyres stated, “Bonneville is surrounded by a low rim, so it was impossible for us to see what was in it from a distance. We have to drive to the rim and look inside” (2005, p. 322). Likewise, Vertesi (2015) recounts the case of scientists seeing different colored bands on the rim of Victoria Crater. They could also sense, however, that they did not have an optimal grip on that feature to determine whether it provided evidence of “layering” of depositional materials. This is because the image was taken from too far away. “Banding” is a more interpretively neutral term that describes a visible phenomenon but not its geological origins, whereas “layering” implies a type of depositional mechanism (e.g., aeoloan or alluvian) that produced the bands. This led to the formation of further P-intentions to get the optimal grip needed to determine whether layering was present; they examined images to determine the best locations from which to take Pancam images to answer the stratigraphic questions.

The ability to identify relevant affordances on the Martian surface through static images required MER team members to use their imaginative skills. According to Gallagher's 2015 enactive account, imagination is a type of pretense, a kind of overt or covert simulated activity. As he says, “We should think of imagination first as a kind of active engagement with possibilities…the imagining just is the playacting. It's literally enacting something in bodily movement that may include the use of props” (p. 193). Imagination can therefore expand the affordance space.

In the case of Mars exploration, team members often imagined that they were the rover acting on the surface of Mars. Experiencing core-presence was thus facilitated by what the ethnographic reports describe as “becoming the rover” (Clancey, 2012; Vertesi, 2012, 2015). It is an example of the sense of embodiment that can arise when working with robots, where operators have the illusory experience that a robot's body is their own body (Toet et al., 2020). “Becoming the rover” involved team members developing an embodied sense of the rover's capacities and then acting them out. Vertesi (2012, p. 400) calls this identification with the rovers “technomorphism,” which involves “developing a sensibility to what the rover might see, think, or feel, in relation to specific activities that must be planned.”

Technomorphism was displayed by engineers on the team. For example, Vertesi (2012) observed a Pancam operator pretending to be a rover by using her hands held up to her head to represent the left and right Pancams, and then rotating at the waist. She did so to work out how the rover would have to move its IDD to image a particular target. MER scientists also displayed technomorphism. This was achieved as a result of their experience requesting images, drives, and measurements, giving them a sense of what the rover body could accomplish, thereby enabling them to use their imagination to identify relevant affordances. Their technomorphism was evident in their use of gestures. While looking at images during SOWG meetings, they enacted the rover, moving the chairs they were sitting on to work out potential movements (Vertesi, 2015). They also used an arm to work out movements of the rover's IDD. As Vertesi says, “this involves lifting the right upper arm to shoulder height, dropping the forearm to 90° with the fist pointed at the ground, and articulating the arm in a limited fashion first side-to-side from the shoulder, then swinging forward from the elbow” (2015, p. 172). When scientists worked out a potential sequence of maneuvers using the IDD, they often used these gestures. There was a correct way to move one's body in simulating the rover that team members knew, and they could recognize team members by whether or not they used these gestures. Being able to enact the rovers' movements provided team members common ground for understanding each other, which facilitated the process of arriving at a consensus regarding shared P-intentions.

Scientists' becoming the rover also influenced their perceptions of which features of the Martian terrain could be interacted with using the specific tools onboard the rovers. Becoming the rover thus enabled them to obtain an optimal grip on a field of relevant affordances in the Martian landscape. For example, scientists often worked with images that distorted visual features, such as images produced by fish-eye lenses. Nonetheless, they often worked with these images in raw form and developed an intuitive sense of the spatial relations within them (Vertesi, 2015). As a result, they came to inhabit the environment in the way that the rover does. What was perceived on images as near vs. far, reachable vs. not reachable, RAT-able vs. not RAT-able, for example, was determined by the constraints and opportunities offered by the rover body, and scientists developed a skillful know-how of these factors. Indeed, scientists' ability to acquire an objective, propositional understanding of the geology of Mars depended on their being able to dwell as the rover and interact with the surface of Mars in a skillful manner.

The enactment of the rover body also affected the emotions of the team members in ways that influenced their action readiness. With practice they acquired a sense of the environmental conditions that would endanger the rover, which had to be kept within narrow limits of, for example, temperature and battery levels to remain operational. It also had to be kept away from very loose soil where it could become stuck, or from steep cliffs where it could fall. Thus, any proposed movement in a direction that would endanger the rover led to an appropriate affective reaction in the team. When looking at images, certain paths looked inviting and others forbidding, depending on the current abilities of the rover. According to Wirth et al. (2012), emotional involvement is crucial for the experience of spatial presence, so it is likely that emotional reactions by MER team members in response to environmental affordances helped to foster a sense of core-presence on the landscape. Indeed, failure to predict interoceptive reactions has been linked to a loss of presence (Seth, 2013).

A plausible explanation for why team members identified with the rovers and why this enhanced their ability to respond to environmental affordances comes from research on the benefits of self-referential information processing (Symons and Johnson, 1997). It shows that processing information relevant to the self serves as “associative glue” that enhances binding in attention, perceptual integration, memory, and decision-making (Sui and Humphreys, 2015). Indeed, fMRI studies have found that compared to other-person processing, self-relevant processing simultaneously leads to enhanced activation of brain regions associated with representations of self and areas associated with the orienting of attention to environmental stimuli (Sui et al., 2013). Due to the rapid binding of information that takes place in self-relevant processing, representations of the self can quickly expand to incorporate other objects. Generally, this “self-expansion” takes place “so that more attributes are available to help an individual attain a goal” (Sui and Humphreys, 2015, p. 726).

In the case of team members embodying the rover, this can enhance their attention regarding features of the Martian surface. As Clancey (2012, p. 110) says, “the projection of the self into the rover is an embodied way of synthesizing…disparate sources of information.” The binding of features of the rover can, however, also produce some strange somatic associations between what is happening to the rover's body and what is happening to the body of individual team members. As Vertesi (2012, p. 402) recounts, one scientist reported the following: “I was working in the garden one day and all of a sudden, I don't know what's going on with my right wrist, I cannot move it—-out of nowhere! I get here [to the planning meeting], and *Spirit* has, its right front wheel is stuck! Things like that, you know?. I am totally connected to [*Spirit*]!” Similarly, another scientist reported the following:

[I]nterestingly, I screwed up my shoulder. and needed surgery on it right about the time that *Opportunity's* IDD [arm] started having problems [with a stiff shoulder joint], and I broke my toe right before *Spirit's* wheel [broke], so I'm just saying, maybe it's kind of sympathetic, I don't know, [laughs] I mean I don't think there's any magic involved or anything but maybe it's some kind of subconscious thing, I don't know (Vertesi, 2012, p. 403).

It is important to point out that the sense of embodiment in the MER mission was acquired in a way that differs from typical cases where operators come to embody their robots. In most situations where it has been demonstrated, a teleoperation mode of control has been employed (Toet et al., 2020). That is, the operator controls a robot by issuing commands through a manual control unit, and receives near real-time feedback from sensors as the remote action is unfolding (Niemeyer et al., 2016). Under these conditions, the proximal tool can be incorporated into the operator's body schema, altering peripersonal space and producing a shift of location (e.g., Riva and Mantovani, 2012; Bourgeois et al., 2014). In MER, however, scientists only indirectly controlled the rover tools, and feedback was significantly delayed, as the results of actions were not available until the following sol. The shift in spatial presence instead arose through the exercise of the embodied imagination. MER scientists imagined they were the rover acting on the surface of Mars and their ability to grasp affordances improved as they were able to successfully anticipate the results of the actions they requested. This led to the rover body being written onto their body schema. Indeed, some research has found that merely imagining using a tool can lead to changes in body schema (e.g., Baccarini et al., 2014). Moreover, as Aymerich-Franch et al. (2015) have shown, operators can embody a robot and experience a shift in location even when feedback is delayed and control over its movement is only partial.

### Proto-Presence on Mars

This level requires a tight coupling between motor responses and specific features of objects. It involves the enactment of M-intentions that provide fine-grained guidance and control. Because M-intentions operate over elemental timescales, they are significantly affected by delays between actions and feedback regarding the consequences of those actions. In the case of the MER mission, although scientists requested specific observations and drives, it was the responsibility of engineers to program the M-intentions sent to *Spirit* and *Opportunity*, and the software on the rovers interpreted these instructions and executed them through the rover hardware. Rover Planners were responsible for programming the rover activities. They used imagery provided by the Navcams and Hazcams on the rovers along with the Rover Sequencing and Visualization Program (RSVP) to develop and test the sequence of commands to be sent each sol (Clancey, 2012). The RSVP software specified particular movements and orientations of the rover arm and its instruments. Scientists identified specific targets and the observations to be carried out on them using the SAP (Science Activity Planner) software, and the RSVP software converted these into specific movements of the rover tools. Although the consequences of the instructions were simulated as the sequence was being developed, precise feedback on the results of the actions was not available until many hours later when data from the rovers was downloaded.

The Rover Planners, however, were not the only ones responsible for determining M-intentions. Instrument experts were also involved. For example, when the scientists wanted to analyze a rock in the Columbia Hills called “Pot of Gold,” they first had to scrape off some of its surface. But, as Squyres (2005) points out, it was not an easy target to RAT, as it could damage the tool due to its shape. The engineers in the RAT team had to figure out the range of acceptable angles from which to apply the instrument. This information was used by the Rover Planners who had to determine the precise direction *Spirit* should approach the rock to place the IDD. Moreover, the rovers themselves had the capacity to enact some M-intentions on their own and to monitor their own actions for unwanted side effects. For example, the rovers could halt their movement if sensors detected that the pitch, roll or tilt exceeded a particular range (Leger et al., 2005). In addition, part way through the mission the *AutoNav* software was uploaded to the rovers, which enabled them to plan their own route because they could use their onboard cameras to autonomously detect obstacles and to select a path around them, formulating M-intentions on the fly. Rover Planners alternated between using and not using this software depending on the situation because using it increased drive times, but freed them to focus on other tasks (Clancey, 2012).

It is important to point out that the scientists did, of course, enact some M-intentions as they went about formulating strategic and tactical plans. These allowed them to carry out the physical/motoric component involved in attending meetings, engaging in discussions with colleagues, working with images, annotating documents, making decisions, and so on. Without the ability to enact the required M-intentions to do these things, they would not be able to achieve their intentions of establishing shared D- and P-intentions. Likewise, engineers exhibited various M-intentions that served to underwrite their goal to create the daily program for the rovers to enact. The M-intentions of scientists and engineers, however, were different from the M-intentions that were enacted by the rovers on the surface of Mars. Those M-intentions, though displaced spatiotemporally from the D- and P-intentions of scientists, were indirectly caused by the scientists' and engineers' higher level intentions, and they served to specify the micro-components needed to actually carry them out. Nonetheless, because it is a distributed cognitive system, scientists and engineers did not experience those sensorimotor contingencies as they unfolded. That is why they lacked proto-presence on Mars. In mundane situations, M-intentions coincide with P-intentions such that a person can simultaneously experience core-presence and proto-presence.

To summarize, there were M-intentions enacted on the surface of Mars, as the rovers were able to skillfully interact with the landscape, responding to affordances present in the Martian environment. Nonetheless, the experience of presence scientists possessed cannot have been due to enacting these M-intentions. This is because they did not experience the precise sensorimotor contingencies themselves. It is the rovers that acted on the surface and it is engineers that programmed these intentions. Moreover, M-intentions typically unfold over an elemental timescale. In the case of Mars exploration, however, when engineers programmed them, it was often many hours before they were to be executed. Thus, there was a significant delay between the formation of an M-intention and feedback regarding its execution.

More generally, batch programming is incompatible with the experience of proto-presence. Within certain parameters, however, it is possible with a teleoperation mode of control. This mode is very sensitive to the lags between when a command is issued and when feedback is received. Indeed, Lester and Thronson (2011) argue that for systems controlled via teleoperation, the upper limit of acceptable delays is about 200 msec. Beyond that, performance drops off and the sense of presence in the remote environment collapses. As a result, multiplying human detectable latencies by the speed of light gives us what they call the “cognitive scale of the universe,” which they regard as the limits of tele-operated extended cognition. For humans, the cognitive scale of the universe is about 30,000 km for latencies of 200 msec. People must be that distance or less to have a sense of proto-presence on Mars. This means proto-presence on Mars through the joysticking control of robots is not possible from Earth, since it is on average 225 million km away.

**Figure 4:**
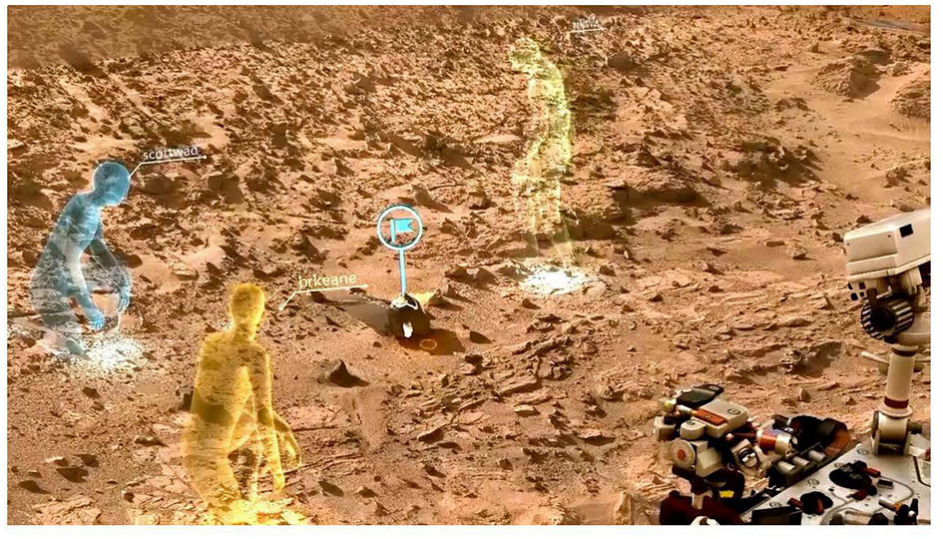
*OnSight* used for visualization on the Mars Science Lab mission. Image credit: NASA/JPL-Caltech.

A move in the direction of proto-presence on Mars, however, is offered by the *OnSight* system (Abercrobie et al., 2017). *Onsight* is a 3D visualization tool developed at the NASA Jet Propulsion Lab for the MSL mission (see Figure 4). It runs on the Microsoft HoloLens headset, providing an immersive view of the terrain around the *Curiosity* rover that people wearing the headset can walk through. *OnSight* has a multi-user feature that allows team members to meet virtually on Mars and even have joint “field trips.” When they wear the headset they can see avatars of each other. Moreover, because *OnSight* tracks where each person is looking, displaying this as a “gaze ray” that projects from the head of their avatar, people are able to see what their colleagues are looking at. They can also annotate features of the landscape and highlight points of interest with flags, which facilitates shared attention (For a demonstration of *OnSight* see https://www.youtube.com/watch?v=XtUyUJAVQ6w).

Research conducted at JPL suggests *OnSight* is used by scientists to increase understanding of features of the Martian terrain, and by engineers to inform vehicle operation. Nonetheless, although *OnSight* has come to be widely used by team members on MSL, it has not fully replaced the 2D and 3D imagery. Indeed, most use of *OnSight* appears to be of short duration (i.e., < 20 min by single users) and most of the work is still carried out using the same visualization tools that were employed during the MER mission. This is likely because the immersive display is of lower resolution than computer monitors (Abercrobie et al., 2017). As a result, although it may be useful for addressing certain scientific questions, such as how certain features are distributed spatially in the Martian landscape, it is not suitable for addressing other types of scientific questions that require higher resolution, like fine details of rock morphology and texture. Moreover, because the surface that team members walk on is different from the Martian ground displayed through the headset, there are sensorimotor inconsistencies that undercut proto-presence on Mars. Scientists also cannot interact with the objects that they see through the headset in ways that they would be able to if they were physically present on Mars. They cannot kick over rocks or pick them up and feel them. This effaces proto-presence because in mundane situations it is a multimodal experience.

## Conclusion and Future Directions

Scientists and engineers working on Mars through rovers in MER reported experiencing a collective presence on the Martian landscape through the rovers. Prima facie, this sense of presence is problematic because neither scientists nor engineers experienced the tight sensorimotor coupling that normally underlies the experience of presence in mundane situations (Chiappe and Vu, 2019). We argue, however, that Riva and Waterworth's 2014 Three-Level model can be used to characterize the experience of presence of team members on the MER mission. In particular, we maintain that although proto-presence was not possible, extended- and core-presence were possible to attain. According to the Three-Level model, presence is a result of successfully enacting intentions at different levels. When there is a successful cascade of intentions, such that D-intentions are successfully translated into P-intentions, and these into M-intentions, a maximal sense of presence is achieved. In the case of Mars exploration, we find all three levels of intention. However, the intentions were shared intentions, reflecting the collective will of the team. Moreover, they were enacted by different elements within a distributed cognitive system. Scientists were only involved in the enactment of D-intentions, which were formulated during strategic planning meetings, and P-intentions formulated during the daily SOWG meetings. The relevant M-intentions, which depend on tight sensorimotor coupling, were programmed by engineers but were only fully enacted by the software and hardware of the rovers.

Importantly, the length of the time involved in enacting D- and P-intentions far exceeded those characteristic of mundane situations, and individuals at times complained of the slow pace of the work. They complained that if they were physically present on Mars, they would be able to do in a few minutes what the rovers take a day or longer to accomplish. Thus, the work environment and the structure of the sociotechnical system involved in Mars exploration likely prevented the sort of flow experience that can accompany certain types of interactions with technology (Triberti et al., 2021). The lengthy timescales were well-suited, however, for participatory knowing, and for establishing a collective sense of presence on Mars by a distributed cognitive system. As Mindell (2015, p. 184) says, “the MER team had a deep sense of presence in the landscape they are studying. ‘*We were all there, together, through a robot!'*.” The feeling of core-presence and extended-presence depended on successfully responding to affordances revealed through images. This was achieved by team members collectively “becoming the rover,” thus absorbing its embodiment with its constraints and abilities.

The model of presence we are advocating for relies on evidence provided by open-ended interviews conducted by participant-observer ethnographers (Clancey, 2012; Vertesi, 2015) and by the firsthand account of the mission PI (Squyres, 2005). As a result, future research will have to be conducted to further validate our account. This includes using the various tools such as questionnaires that require participants to self-report on their sense of spatial presence (see Grassini and Laumann, 2020 for an overview) and on their sense of embodying remote tools (e.g., Kilteni et al., 2012). The metrics that need to be used may have to be modified to explore the different types of presence referred to by the Three-level model. Furthermore, due to the challenges of accessing the population of interest, it is likely that much of the work to validate our account will have to be done using suitable lab-based analogs. This is not unheralded, as much research in Human Factors is carried out this way. For example, simulations are used to study the factors that influence how air traffic controllers maintain situation awareness and manage their workload (e.g., Chiappe et al., 2016). Indeed, we hope that by developing suitable metrics and lab-based techniques we will be able to offer a general account that will serve to illuminate how presence is achieved by distributed cognitive systems working with complex technology in distant locations. This understanding can facilitate the development of tools that make core-presence and extended- presence possible in a wide range of domains.

## Data Availability Statement

The original contributions presented in the study are included in the article/supplementary material, further inquiries can be directed to the corresponding author/s.

## Author Contributions

DC and JV contributed equally to the development of the arguments contained in the paper. DC wrote the first draft of the manuscript and its revision. JV provided edits and helped get it ready for submission. Both authors contributed to the article and approved the submitted version.

## Conflict of Interest

The authors declare that the research was conducted in the absence of any commercial or financial relationships that could be construed as a potential conflict of interest.

## Appendix

### The suite of MER rover tools

Panoramic cameras (Pancams): Two mast-mounted cameras that provide color images in stereo with 13 filters.

Navigation cameras (Navcams): Two mast-mounted black and white cameras that are used to image terrain in stereo for planning drives and deploying other instruments.

Hazard cameras (Hazcams): Mounted on lower portion of the rover, two in front and two in rear, to image potential obstacles in black and white, with fish-eye lenses.

Miniature Thermal Emission Spectrometer (Mini-TES): Mast-mounted spectrometer that identifies mineral composition at a distance.

Microscopic Imager (MI): Located on the rover arm (the Instrument Deployment Device, IDD) and is a combination camera and microscope.

Alpha-Particle X-Ray Spectrometer (APXS): Detects elements by bombarding rocks with alpha rays and x-rays, and detecting the energy of these particles as they bounce back from the surface of the rock.

Mössbauer spectrometer (MB): IDD mounted spectrometer that uses gamma rays to determine the composition and quantity of iron-bearing minerals on rock surfaces.

Rock abrasion tool (RAT): IDD mounted tool with rotating teeth that grinds holes onto the surface of rocks.
